# Poor Iodine Knowledge, Coastal Region, and Non-Iodized Salt Consumption Linked to Low Urinary Iodine Excretion in Zhejiang Pregnant Women

**DOI:** 10.3390/nu11020413

**Published:** 2019-02-15

**Authors:** Xiaofeng Wang, Xiaoming Lou, Zhe Mo, Mingluan Xing, Guangming Mao, Wenming Zhu, Yuanyang Wang, Yuan Chen, Zhifang Wang

**Affiliations:** Department of Environmental Health, Zhejiang Provincial Center for Disease Control and Prevention, 3399 Binsheng Road, Hangzhou 310051, China; xfwang@cdc.zj.cn (X.W.); xmlou@cdc.zj.cn (X.L.); zhmo@cdc.zj.cn (Z.M.); mlxing@cdc.zj.cn (M.X.); gmmao@cdc.zj.cn (G.M.); wmzhu@cdc.zj.cn (W.Z.); yywang@cdc.zj.cn (Y.W.); yuanchen@cdc.zj.cn (Y.C.)

**Keywords:** iodine, knowledge, iodine status, iodized salt, pregnancy

## Abstract

Background: Iodine deficiency in pregnant women, defined as a median urinary iodine concentration (UIC) of less than 150 μg/L, is an important public health issue. To improve their iodine intake, it is important to understand the knowledge and practices regarding iodine. Methods: A cross-sectional investigation was conducted on 2642 pregnant women during 2016–2017 in Zhejiang province, China. A 3-point Likert scale questionnaire was used to record knowledge. The UIC and iodine content in household salt were determined. Results: Coastal participants were iodine deficient (median UIC 127.6 μg/L) while inland participants were iodine sufficient (median UIC 151.0 μg/L). The average knowledge scores were significantly lower for the coastal participants (24.2 points vs. 25 points for the inland participants; *p* < 0.001). The percentage for iodized salt consumption was significantly lower for the coastal participants (88.9% vs. 96.0% for those inland; *p* < 0.001). A generalized linear model analysis showed that non-iodized salt consumption, coastal region, and low knowledge scores were independently associated with a low UIC. Conclusions: Comprehensive interventional strategies are needed to develop to achieve an optimal iodine status. We recommend that coastal pregnant women should take iodine supplements based on the consumption of iodized salt, and improvement of iodine-related knowledge.

## 1. Introduction

Iodine is an essential micronutrient in the human body for the synthesis of thyroid hormones, which control and regulate metabolism. A diet lacking iodine may result in iodine deficiency disorders (IDD). Pregnant women are the most vulnerable population to IDD since they have an increased need for iodine compared to nonpregnant women. IDD in pregnancy has adverse effects on fetal health. The most destructive problems induced by IDD in pregnancy are retarded physical growth and brain development. Overt endemic cretinism is the consequence of severe IDD in pregnancy.

The median urinary iodine concentration (UIC) is recommended by the World Health Organization (WHO) as an important indicator to assess iodine nutrition in pregnant women [1]. The first and largest national IDD surveillance for pregnant women was conducted in 2014. A total of 19,500 pregnant women from 31 provinces of China was sampled via probability proportional to size (PPS) in this surveillance survey. The results showed that the median UIC was 154.6 μg/L [2], indicating that the pregnant population was iodine sufficient at a country level based on the WHO-recommended criterion of the lowest cut-off value for optimal iodine nutrition (150 μg/L) [1]. However, recent surveillance studies have shown that the distribution of iodine nutrition in Chinese pregnant women displays inter- and intra-provincial differences, due to factors such as different geographical residence characteristics [3,4,5] and dietary habits [5]. Pregnant women identified with iodine deficiency were mainly from the coastal provinces of China [2,6]. Deficient iodine nutrition in pregnancy is therefore of new public health importance in these provinces, since mild IDD in pregnancy may impair cognitive outcomes in infancy [7,8,9].

Previous studies have shown that low levels of iodine-related knowledge and practices are correlated with a risk of low UIC [10,11,12,13]. However, studies about the association between the iodine nutrition status among pregnant women and their knowledge and practices are still sparse in China. We therefore conducted this research to describe pregnant women’s iodine-related knowledge and practices and to explore the potentially influential factors on iodine deficiency in pregnancy.

## 2. Materials and Methods

This cross-sectional survey was conducted between March 2016 and June 2017 in the Zhejiang province of China, as shown in our previous study [14], including both the coastal and the inland regions.

A multistage stratified random sampling was performed. This sampling method was broadly in accordance with China’s national IDD surveillance protocol [2] and the WHO guidance on monitoring iodine nutrition status in a pregnant population [1]. Based on the lengths of coastline, we first categorized a total of 11 city-level administrative divisions of Zhejiang province into two regions—the coastal region (Zhoushan, Taizhou, Wenzhou, Ningbo, Hangzhou, Jiaxing, Huzhou, and Shaoxing cities) and the inland region (Jinhua, Quzhou, and Lishui cities). Second, we selected all counties within these two areas. Third, 100 pregnant women attending antenatal care at community health centers from each selected county were recruited in both the coastal and the inland regions, after adjustment for age and trimester.

A 10 mL morning spot urine sample and a 30 g household cooking salt sample were collected from each participant. The inductively coupled plasma mass spectrometry (ICP-MS) method and the titrimetric method were taken as the reference methods for the determination of UIC and salt iodate content, respectively. In this epidemiological study, UIC was measured using the WHO-recommended As^3+^-Ce^4+^ catalytic spectrophotometry method with ammonium persulfate digestion (WS/T 107.1-2016), based on the Sandell-Kolthoff reaction. The standard deviation (SD) for the precision of this method was 1.2%–1.7% and the recovery of added iodine was 98.6%. The household salt iodate concentration was measured using the iodometric titration method with thiosulphate, recommended by the WHO and China (GB/T 13025.7-2012). All samples were determined in China’s National Iodine Reference Laboratories, which participated in an internal quality control program that was in place and an external quality assurance program run by the National Center for Disease Control and Prevention of China (CDC).

A questionnaire assessing participants’ iodine-related knowledge was adapted from previous studies [10,13,15,16] and then adopted after three experts reviewed its clarity and comprehension and agreed on all the items used in the questionnaire. The reliability coefficient (alpha) of the questionnaire was 0.843, which was considered acceptable. It was a 3-point Likert scale questionnaire consisting of 10 items (see Supplementary Questionnaire). Participants were asked about their knowledge on the following items: (1) the necessity for iodine (Question 1), (2) the adverse fetal health effects associated with IDD in pregnancy (Questions 2 and 3), (3) the prevention of IDD in pregnancy (Questions 4 and 5), (4) the meaning of the logo on iodized salt packages (Question 6), (5) the necessity of iodized salt consumption (Questions 7 and 8), (6) the increased requirement for iodine in pregnancy (Question 9), and (7) the current iodine nutrition status in Zhejiang pregnant women (Question 10).

Knowledge variables were calculated as total knowledge scores for each participant. A correct answer generated 3 points, while “Don’t know” and incorrect answers were given 2 points and 1 point, respectively. In this study, the iodine nutrition status in pregnancy was assessed via the median UIC, according to the recommended level given by the WHO [1] and the United Nations International Children’s Emergency Fund (UNICEF) [17]. Briefly, the sampling error (95% confidence interval (CI) of the median UIC) was considered and calculated using bootstrapping. When the 95% CI included the WHO-recommended lowest cut-off value of optimal iodine status (150 μg/L), the iodine status in pregnancy was considered optimal. When 150 μg/L was higher than the upper cut-off level of the 95% CI, the iodine status was considered deficient. Household cooking salt was classified into non-iodized salt (<5 ppm iodine) and iodized salt (≥5 ppm iodine).

All participants gave their informed consent before they participated in this study. This research was conducted following the Declaration of Helsinki, and the protocol was approved by the Ethics Committee of Zhejiang Provincial CDC (ZJCDC-20160302).

Epi Data version 3.1 software (EpiData Association, Odense, Denmark) was used to input data. Double data entries were performed. IBM SPSS version 23 software (IBM Corp., Armonk, NY, USA) was used for all analyses. Only data on those pregnant women who had a known singleton pregnancy or did not report a history of thyroid disorders or other chronic diseases were further treated for final analysis. Count data were expressed as numbers and percentages (%). Comparisons of the count data were performed via chi-square tests. The Kolmogorov-Smirnov test was used for normality. The mean and SD were used to describe the normal variables, while the median and 95% CI were used for the non-normal variables. The Cochran-Armitage test for trend was performed to test the linear-by-linear trend between the percentage of participants’ iodized salt consumption and their knowledge scores and between iodine nutrition and knowledge scores. Binary logistic regression analyses were used to explore the potentially influential factors of knowledge on low UIC, and a generalized linear model was chosen to explore the interactive effects on UIC. A *p* value below 0.05 was determined to be statistically significant.

## 3. Results

### 3.1. Sociodemographic Characteristics of the Participants

Table 1 shows the sociodemographic characteristics of all 2642 participants in the inland and coastal regions. A significantly lower percentage of participants living on the coast (45.7%) were homemakers when compared with those inland (63.7%; *p* < 0.001). The percentage of participants residing in urban areas was significantly higher on the coast (66.3%) than inland (34.9%; *p* < 0.001). There were significant differences in higher education (high school and above) and high income (≥10,000 USD) defined by region—65.0% on the coast vs. 55.3% inland and 17.9% on the coast vs. 6.7% inland, respectively (both *p* < 0.001). No other significant differences in sociodemographic characteristics (age, trimester, ethnicity, and cigarette smoking status) were defined among participants between these two regions (all *p* > 0.05).

### 3.2. Iodine-Related Knowledge

The data regarding iodine-related knowledge used to calculate the total knowledge scores are presented in Table 2. The range of the total iodine-related knowledge scores was between 16 and 30, with the 25th, 50th, and 75th percentiles at 19, 23, and 27 points, respectively. Participants’ knowledge was then categorized as follows: ≤19, 20–23, 24–27, and 28–30 points.

The average knowledge score in the coastal regions (24.2 ± 2.7) was significantly lower than that inland (25.0 ± 2.9; *p* < 0.001). The percentages of participants with 24–27 and 28–30 points were significantly lower on the coast (46.0% and 17.2%, respectively) than inland (53.8% and 20.1%, respectively; both *p* < 0.05). However, the percentages of participants with ≤19 and 20–23 points were significantly higher on the coast (4.8% and 32.0%, respectively) than inland (2.3% and 23.8%, respectively; both *p* < 0.05; Table 2).

Regarding the four sections of knowledge questions (iodine, IDD, iodized salt, and iodine nutrition), the coastal participants were generally more likely to respond with incorrect answers when compared with the inland participants (Table 3). The percentage of coastal participants understanding that “the iodine element is essential to humans” (85.8%) was significantly higher than that of the inland participants (81.2%; *p* < 0.001). However, the coastal participants showed a deep lack of knowledge of the health effects of IDD, iodized salt, and iodine nutrition. On average, the percentage of coastal participants with correct answers (58.6%) was nearly three percentage points lower than that of inland participants (61.8%). Significantly lower percentages of coastal participants were aware that “IDD in pregnancy has adverse effects on fetal brain development and growth” (69.9% and 72.0%, respectively) when compared with 75.9% and 77.7% of the inland participants (both *p* < 0.05). In terms of the question on “what is the most efficient method to prevent IDD”, only 66.6% of the coastal participants chose “consuming iodized salt”, which was significantly lower than the proportion of inland participants who gave the same response (73.4%; *p* < 0.001). Meanwhile, more participants from the coastal regions (14.6%) mistook “consuming seafood” for an effective means of preventing IDD, in comparison with 7.0% of those from the inland regions (*p* < 0.001). When it came to iodized salt consumption, participants on the coast were more likely to have the wrong ideas that “I do not need to consume iodized salt” (54.5%) and “I am able to meet my iodine requirement when following a diet containing enough seafood but no iodized salt” (22.5%), compared to 44.6% and 18.8% of inland participants (both *p* < 0.05). A significantly higher percentage of participants who considered their iodine nutrition “excessive” was found on the coast (26.9% on the coast vs. 23.5% inland; *p* < 0.05). No other differences in knowledge about preventing IDD, the logo on the salt package, and the increased iodine requirement in pregnancy were identified between these two regions (all *p* > 0.05).

### 3.3. Behavior of Consumption of Iodized Salt and Its Correlation with Knowledge Scores

**Figure 1:**
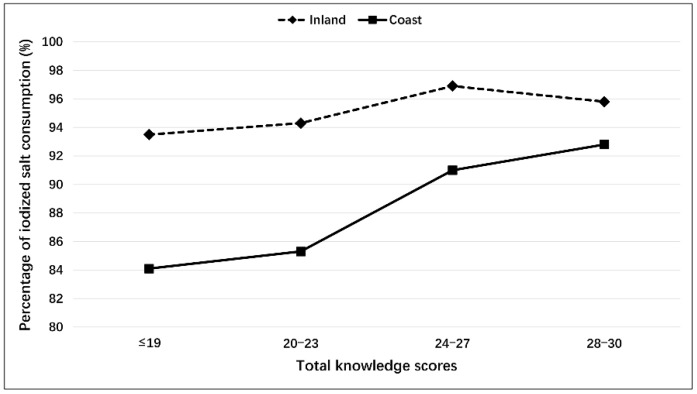
Percentage of iodized salt consumption according to knowledge scores and region.

The median iodine concentration in salt was 23.7 ppm (95% CI 23.2–23.5 ppm) on the coast, which was not significantly different from that inland (23.6 ppm, 95% CI 23.3–23.6 ppm; *p* = 0.552). The percentage of participants consuming iodized salt was significantly lower on the coast (88.9%) than inland (96.0%; *p* < 0.001). Figure 1 shows that the percentage of participants consuming iodized salt varied according to their knowledge scores and region. For those participants living on the coast, there was a significant upward trend in the consumption of iodized salt with an increased knowledge score (*p*_linear-trend_ < 0.001). The percentage gradually increased from 84.1% in those with scores of ≤19, to 85.3% in those with scores of 20–23, to 91.0% in those with scores of 24–27, and then to a peak of 92.8% in those with scores of 28–30. For the participants residing in the inland region, however, when their knowledge scores increased from ≤19 to 28–30, the percentage of iodized salt consumption did not show a significant change (*p*_linear-trend_ = 0.205), instead it stably fluctuated between 93.5% and 96.9%.

### 3.4. Iodine Nutrition Status and Its Correlation with Knowledge Scores and Iodized Salt Consumption

The median UIC was 151.0 μg/L (95% CI 144.6–157.7 μg/L) for the inland participants and 127.6 μg/L (95% CI 123.4–133.6 μg/L) for the coastal participants. Those participants in the inland region were categorized as having iodine sufficiency, while those in the coastal region were classified as having iodine deficiency, according to the iodine nutrition assessment criterion recommended by the WHO [1] and the UNICEF [17]. A nonparametric test also showed that iodine nutrition in pregnancy was distributed with a regional difference (*p* < 0.001).

**Figure 2:**
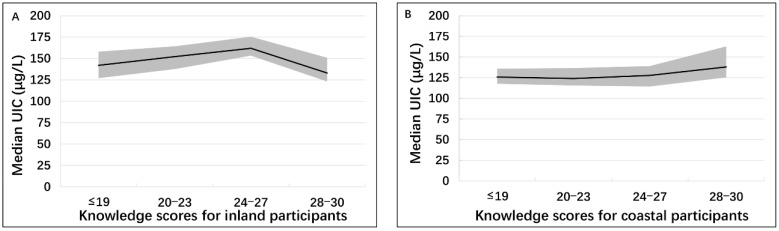
Urinary iodine status distributed by the total knowledge scores among participants in the inland region (**A**) and the coastal region (**B**). Black lines show the median urinary iodine concentration (UIC). Light gray shadows show the corresponding 95% confidence interval (CI) of the median UIC.

The distribution of iodine nutrition by region and knowledge score is found in Figure 2. For participants living inland, no significant difference was found between iodine nutrition and knowledge scores (*p*_linear-trend_ = 0.579). Each 95% CI of the median UIC corresponding to the knowledge scores included the WHO-recommended optimal level of 150 μg/L, indicating that the inland participants with different knowledge scores were all iodine sufficient. However, for those living on the coast, the 95% CI (125.3–162.8 μg/L, median 138 μg/L) included the WHO-recommended level of 150 μg/L only when their knowledge scores reached the highest level (28–30 points). For those with scores lower than 27, each 95% CI of the median UIC did not include the level of 150 μg/L: 117.7–135.9 μg/L for scores of ≤19 (median 125.8 μg/L), 115.6–136.7 μg/L for scores of 20–23 (median 123.9 μg/L), and 114.3–139.0 μg/L for scores of 24–27 (median 127.8 μg/L). This indicates that in the coastal region, the participants with 28–30 points were iodine sufficient, while those with scores lower than 27 were iodine deficient (*p*_linear-trend_ = 0.389).

**Figure 3:**
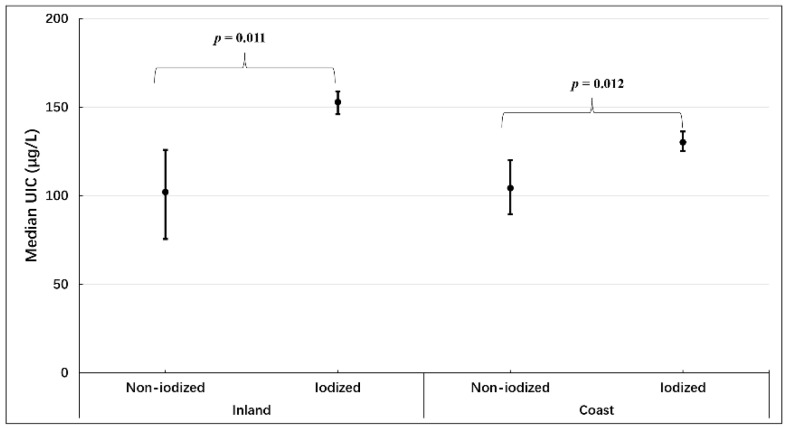
Urinary iodine status among participants according to region and type of salt consumed.

The distribution of iodine nutrition among participants, according to type of salt consumed and region, is found in Figure 3. For the inland participants, those consuming iodized salt were iodine sufficient (median 152.9 μg/L, 95% CI 145.6–159.0 μg/L), whereas those consuming non-iodized salt were still iodine deficient (median 102.2 μg/L, 95% CI 75.6–125.9 μg/L; *p* = 0.0012). For the coastal participants, those consuming iodized salt remained iodine deficient (median 130.2 μg/L, 95% CI 125.3–136.3 μg/L), though their UIC was significantly increased in comparison with that of those using non-iodized salt (median 104.3 μg/L, 95% CI 89.6–120.0 μg/L; *p* = 0.011).

### 3.5. Influential Factors of Low UIC

The potentially influential factors of knowledge on low UIC among pregnant women were analyzed via binary logistic regression. The results showed that a response of “consuming seafood” as the best method to prevent IDD corresponded to the highest probability for the participant to have a low UIC (odd ratio (OR) 2.018, 95% CI 1.527–2.668), among all the given answers to questions on iodine-related knowledge (Supplementary Table S1).

We further established the effects of knowledge scores, sociodemographic characteristics (residency, geographical location, occupation, education, and income), and consumption of iodized salt on low UIC through generalized linear model analysis (*p* < 0.001). The factors of consumption of non-iodized salt (*p* = 0.027), coastal region (*p* < 0.0001), and low knowledge scores (*p* < 0.001) were independently associated with low UIC. No other independent association with sociodemographic characteristics was determined (all *p* > 0.05; Supplementary Table S2). Further detailed analysis showed that there was a significant interaction effect among the type of salt consumed, geographical location, and knowledge score (*p* = 0.003). In the coastal region, those participants with a high level of iodine knowledge (28–30 points) and consuming iodized salt were iodine sufficient (median 139.0 μg/L, 95% CI 125.4–152.7 μg/L), whereas those with low knowledge scores (≤27 points) and consuming non-iodized salt were iodine deficient (median 107.3 μg/L, 95% CI 91.0–132.2 μg/L). Similar effects of knowledge and the type of salt consumption on UIC also appeared in the inland population, with iodine sufficiency in those having a high level of knowledge and consuming iodized salt (median 137.7 μg/L, 95% CI 125.4–152.7 μg/L) and iodine deficiency in those showing a low level of knowledge and consuming non-iodized salt (median 81.2 μg/L, 95% CI 67.6–119.1 μg/L).

## 4. Discussion

IDD has been the most easily preventable cause of brain lesions, since universal salt iodization (USI) was introduced. Our previous study showed that Zhejiang pregnant women are mild–moderately iodine deficient, representing approximately 700,000 newborns that are unprotected from the irreversible neurodevelopment damage associated with IDD [5]. Even mild IDD in pregnancy may be associated with adverse effects on the resulting child’s cognitive development. Correcting IDD in pregnancy is therefore essential.

Studies on knowledge and practices related to iodine are helpful to determine the existing nutrition problem, identify the barriers to dietary practices, and feed into the future design of intervention and education [12,13]. To our knowledge, this is the first study assessing and exploring the knowledge, practices, and nutrition related to iodine in pregnant women in China, with a focus on geographical variation. The present study has shown that pregnant women in the coastal region are iodine deficient (median 127.6 μg/L, 95% CI 123.4–133.6 μg/L), while those in the inland region are iodine sufficient (median 151.0 μg/L, 95% CI 144.6–157.7 μg/L), providing evidence that iodine nutrition among Zhejiang pregnant women displays a geographical difference. This result is consistent with that of our previous study [14]. This variability in geography may be explained by three factors. First, the policies implemented to combat iodine-induced diseases via the USI program changed before and after the achievement of eliminating IDD. Historically, nearly 20.7%–45.7% of the Zhejiang population was afflicted with endemic goiter in the 1980s. Zhejiang was hence considered a severely iodine-deficient area. Salt containing 62–260 ppm of iodine was consumed at a household level. With the incidence of endemic goiter decreased to less than 10%, iodine content in salt was set to at least 30 ppm in 1995. During this period of combating IDD, government officers and health care workers worked together to provide iodine-related information, resulting in the rate of iodized salt consumption increasing to more than 95%. After the goal of eliminating IDD was achieved in 2011, concerns regarding the USI program shifted to prevent excessive iodine nutrition, since IDD surveillance results showed that the general population had a greater-than-required iodine intake [18]. Since then, the government’s vigilance on preventing IDD has slipped. The annual financial fund for the elimination of IDD from the government dropped sharply from 15,000 USD to nil. Meanwhile, an increased incidence of thyroid disorders in the coastal provinces [19,20,21,22,23] was documented as associated with a high intake of iodized salt; this was done by endocrinologists who were more likely to analyze the data based only on the patients’ medical records from hospitals, rather than a representative sample from the community. In the above studies, Berkson’s bias inevitably appeared, and the reported incidence of thyroid diseases may be exaggerated. However, this bias was ignored when it came to nutritional education. Finally, the negative idea that the coastal population would have an excessive iodine intake based on consuming daily seafood and iodized salt limited or impeded the provision of correct iodine-related information. The low profile of iodine in China’s public health issues after 2011 may have contributed to a lack of knowledge in the coastal population even though they had a high level of education. A lack of knowledge in the coastal population may be an important factor in their low urinary iodine excretion. Although those coastal participants with a high level of knowledge (28–30 points) were iodine sufficient (the 95% CI of the median UIC included 150 μg/L), those with a low level of knowledge (≤27 points) remained iodine deficient (the upper value of the 95% CI of the median UIC was below 150 μg/L), as shown in this study. Previous studies conducted in developed countries also showed that pregnant women’s knowledge about iodine was positively associated with their UIC. In Norway, where the median UIC among pregnant women was 95 μg/L, 50% of them knew the importance of iodine and only 20%–30% of them understood the main sources of dietary iodine, health effects on IDD, and iodine status [10]. In Australia, where the median UIC in pregnancy was 87.5 μg/L, only about 35% knew the importance of iodine in pregnancy [11]. Similarly, low levels of iodine knowledge were reported in UK women whose median UIC was only 50 μg/L [15]. Therefore, there is a clear need for closing the iodine information gap among coastal pregnant women in the Zhejiang province. Further research is needed to explore the preferred, effective option for information sources.

Second, in coastal areas where a large amount of seafood is available due to the region’s proximity to the ocean, seafood is a minor dietary iodine source, contributing only about 10% of daily iodine intake, whereas iodized salt remains the primary dietary iodine source and contributes to at least 70% of daily iodine intake [24,25]. This present study showed that the percentage of coastal pregnant women using iodized salt (88.9%) was significantly lower than that being used inland (96%) and less than the national lowest cut-off value for the elimination of IDD of 95% (GB 16006-2008). These results indicated that the decreased level of iodized salt consumption might be another important factor that contributes to iodine deficiency. Similar results have been reported in Shanghai city and the Shandong province of China [2], where a lower than 95% consumption of iodized salt (85.6% in Shanghai and 91.4% in Shandong) also resulted in iodine deficiency in pregnant women (126.5 μg/L vs. 144.4 μg/L, respectively). The lack of iodine in the environment has hardly changed, and inhabitants continuously need iodine through iodized salt consumption to maintain euthyroid and a healthy iodine balance. The experiences relating to the progress of the USI program in Vietnam and Ethiopia have proved that the breakdown of USI can result in IDD re-emergence [26,27]. Therefore, the proportion of households using iodized salt should be up to at least 95% before and after the achievement of the elimination of IDD. More urgent efforts are needed to increase the percentage of iodized salt consumption.

Third, the recent decreased iodine content in household salt may be another factor contributing to low urinary iodine excretion in pregnancy. In 2011, the Zhejiang provincial government amended the salt iodine content from 30 ppm to 25 ppm to correct the more-than-adequate iodine nutrition in the general population. With the modified iodine content in salt, the median UIC in both coastal and inland pregnant women decreased by nearly 30 μg/L, from the previously optimal levels of 155.0 μg/L [25] and 187.7 μg/L, respectively, [25] to the currently deficient levels of 127.6 μg/L and 151.0 μg/L, respectively, as shown in this study. Existing data also showed that the median UIC of pregnant women decreased by an average of 20 μg/L at the country level [18]. The geographical difference in iodine nutrition may be attributed to different dietary habits featuring a seafood-heavy diet and a low consumption of iodized salt in the coastal population, but an ordinary diet and a high consumption of iodized salt in the inland population. Based on the critical aspects related to iodine nutrition (geographical location and dietary habits) that have been considered, it seems that it is a challenge to have a single standard of iodine content in salt within one province to ensure that all susceptible populations achieve an optimal iodine status. Although more research is needed to clarify this problem, we suggest that more efforts should be made to achieve optimal iodine nutrition in pregnancy and that geographical differences and dietary habits should be taken into account. For example, apart from iodized salt, coastal pregnant women can get the iodine they need through iodine-containing supplements, which have been widely used in developed countries where mandatory salt iodization has been implemented but iodine requirements in pregnancy are still not met by consuming iodized salt [28,29,30].

Besides this, we found that a substantial portion of the coastal participants were frequently confused between iodized salt and seafood and gave an overestimation of their iodine nutrition. They often mistakenly replace iodized salt with seafood, a potential result of the population’s misconception that they take in too much iodine via the consumption of both iodized salt and seafood [22,31,32]. Consuming seafood containing a varied iodine content may result in unsustainable amounts of iodine in the food supply. However, salt iodization has been proven to deliver a predicted level of dietary iodine. Therefore, educational intervention, especially focusing on the importance of the universal consumption of iodized salt, should be considered.

## 5. Conclusions

This study adds to the increasing evidence that coastal pregnant women in Zhejiang are iodine deficient, particularly those without a good knowledge of iodine and consuming non-iodized salt. An additional complementary strategy for improving iodine intake (e.g., taking iodine-containing supplements) should be recommended to correct the current iodine status in coastal pregnant women, besides the continuous implementation of USI and educational intervention with iodine information. Our finding of a regional difference in iodine status may not be extended to a population with a different iodine status in other provinces of China. To eliminate IDD in pregnancy via a more scientific and precise approach, we suggest that future studies should collect data on dietary iodine habits and clarify the underlying changes in the regional differences of iodine nutrition.

## Figures and Tables

**Table 1:** Participants’ sociodemographic characteristics according to region.

Variables	Inland, *N* (%)	Coast, *N* (%)	*p*
Age (years old)	30.4 ± 5.2	29.9 ± 5.1	0.053
Age groups (years old)			0.999
15–24.9	194 (14.7)	194 (14.7)	
25–34.9	869 (65.8)	869 (65.8)	
≥35	258 (19.5)	258 (19.5)	
Gestational age	21.2 ± 8.4	21.2 ± 8.7	0.947
Trimester			0.999
T1 (0–12 weeks)	250 (18.9)	250 (18.9)	
T2 (13–27 weeks)	692 (52.4)	692 (52.4)	
T3 (≥28 weeks)	379 (28.7)	379 (28.7)	
Ethnicity			0.096
Han	1265 (95.8)	1281 (97.0)	
Others	56 (4.2)	40 (3.0)	
Area of residency			<0.001
Urban	461 (34.9)	876 (66.3)	
Rural	860 (65.1)	445 (33.7)	
Occupation			<0.001
Management and professional	305 (23.1)	549 (41.6)	
Business, service, farming, and fishing	174 (13.2)	168 (12.7)	
Homemaker	842 (63.7)	603 (45.7)	
Highest education level			<0.001
Primary school and below (≤6 years)	79 (6.0)	72 (5.5)	
Middle school (7–9 years)	511 (38.7)	391 (29.6)	
High school (10–12 years)	361 (27.3)	306 (23.2)	
College and above (≥13 years)	370 (28.0)	552 (41.8)	
Income (USD)			<0.001
<5000	322 (24.4)	215 (16.3)	
5000–9999	612 (46.3)	497 (37.6)	
10,000–14,999	299 (22.6)	372 (28.2)	
≥15,000	88 (6.7)	237 (17.9)	
Smoking cigarette status			0.095
Yes	7 (0.5)	2 (0.2)	
No	1314 (99.5)	1319 (99.8)	
Total	1321 (100)	1321 (100)	

**Table 2:** Pregnant women’s total knowledge scores according to region.

Total Knowledge Scores	Inland, *N* (%)	Coast, *N* (%)	*p*
≤19 scores	31 (2.3)	63 (4.8)	0.001
20–23 scores	314 (23.8)	423 (32.0)	<0.001
24–27 scores	711 (53.8)	608 (46.0)	<0.001
28–30 scores	265 (20.1)	227 (17.2)	0.010
Mean ± SD (scores)	25.0 ± 2.9	24.2 ± 2.7	<0.001

**Table 3:** Participants’ iodine-related knowledge according to region.

Knowledge	Inland (*N*, %)	Coast (*N*, %)	*p*
1. Is the iodine element essential to humans?			
Yes◀	1072 (81.2)	1134 (85.8) *	0.001
No	78 (5.9)	27 (2.0) **	<0.001
Don’t know	171 (12.9)	160 (12.1)	0.577
2. Does IDD in pregnancy have adverse effects on fetal brain development?			
Yes◀	1003 (75.9)	924 (69.9) *	0.001
No	24 (2.6)	40 (3.0)	0.479
Don’t know	284 (21.5)	357 (27.0) *	0.001
3. Does IDD in pregnancy have adverse effects on fetal growth?			
Yes◀	1026 (77.7)	951 (72.0) *	0.001
No	24 (1.8)	26 (2.0)	0.710
Don’t know	271 (20.5)	344 (26.0) *	0.003
4. Can IDD be prevented?			
Yes◀	1095 (82.9)	1062 (80.4)	0.173
No	19 (1.4)	20 (1.5)	0.762
Don’t know	207 (15.7)	239 (18.1)	0.132
5. What is the most efficient method to prevent IDD?			
Consuming iodized salt◀	969 (73.4)	880 (66.6) **	<0.001
Consuming seafood	93 (7.0)	193 (14.6) **	<0.001
Others	259 (19.6)	248 (18.8)	0.582
6. What does the logo on the salt package indicate?			
Nonsense	28 (2.1)	19 (1.4)	0.312
Salt has added iodine◀	919 (69.9)	905 (68.5)	0.402
Don’t know	374 (28.3)	397 (30.1)	0.252
7. Do you need to consume iodized salt?			
Yes◀	279 (21.1)	216 (16.4) *	0.002
No	589 (44.6)	720 (54.5) **	<0.001
Don’t know	453 (34.3)	385 (29.1) *	0.004
8. Are you able to meet your iodine requirement when following a diet containing enough seafood but no iodized salt?			
Yes	249 (18.8)	297 (22.5) *	0.021
No◀	807 (61.1)	801 (60.6)	0.225
Don’t know	265 (20.1)	223 (16.9) *	0.035
9. Do women in pregnancy need more iodine than in nonpregnancy?			
Yes◀	717 (54.3)	691 (52.3)	0.334
No	253 (19.2)	289 (21.9)	0.083
Don’t know	351 (26.6)	341 (25.8)	0.658
10. Is the current iodine nutrition in Zhejiang pregnant women excessive?			
Yes	311 (23.5)	356 (26.9) *	0.044
No◀	524 (39.7)	541 (41.0)	0.500
Don’t know	486 (36.8)	424 (32.1) *	0.011

◀: correct or desired answer. *: *p* < 0.05. **: *p* < 0.001.

## Supplementary Materials

The following are available online at http://www.mdpi.com/2072-6643/11/2/413/s1: File S1: Questionnaire on Iodine-Related Knowledge and Practice among Pregnant Women; Table S1: Influential factors of knowledge on low UIC among pregnant women via a binary logistic regression; and Table S2: Influential factors of sociodemographic characteristics, knowledge, and type of salt consumed on low UIC among pregnant women via a generalized linear model.
