# Supplementary material for: Poor Iodine Knowledge, Coastal Region, and Non-Iodized Salt Consumption Linked to Low Urinary Iodine Excretion in Zhejiang Pregnant Women
Source: Nutrients. 2019 Feb 15;11(2):413. doi: 10.3390/nu11020413 (PMC6412776; doi:10.3390/nu11020413)
Supplement: Supplementary file 1 [file nutrients-11-00413-s001.zip › Supplementary Materials/Questionnaire.docx]

**Questionnaire on Iodine-related Knowledge and Practice among Pregnant Women**

**Introduction**

Good morning/afternoon, Mr/Mrs ________.

We are from County Center for Disease Control and Prevention (CDC). We are working on a project related with iodine in which you could participate. The objective of this study is to assess the iodine-related knowledge and practices among pregnant women. The interview will take about 5‒10 minutes. All the data we obtain will remain strictly confidential. Your private information will never be revealed. Meanwhile, you are not obliged to answer any question you would not like to, and you may come to an end of the interview at any time.

The aim of this survey is not to evaluate or criticize you, so please do not feel pressured to if you do not know the answer to any question. I am not expecting you give a specific answer; I would like you to answer questions honestly, telling me about what you know, how you feel, the way you live and how you eat. Feel free to answer questions at your own pace.

Do you agree to participate in this interview?

Yes ___ No ___ If yes, continue to the next question; if no, stop the interview.

Do you have any question before we start? (Answer questions).

May I start now?

Code: Name：

##### Part I. Demographical characteristics:

1. Date of birth: ( based on the ID card)

2. Occupation:

(1) a government official or a cadre of enterprise

(2) a professional staff (e.g. a teacher, a doctor, an engineer and so on)

(3) a Commercial staff

(4) a farmer or a member of the working class

(5) a homeworker

(6) other

3. The highest educational level:

(1) None (<1 year); (2)Primary school (1-5 years); (3) Middle school (6-8 years); (4) High school (9-11years); (5) College and higher (≥12 years)

4. Household net income per capita in 2015: RMB.

(1) ≤29,000; (2) 30,000-59,000; (3) 60,000-99,000; (4) 100,000 and above

5. Gestation period: weeks

6. Gravidity: (1) Primigravida (2) Multigravida (please specify )

7. Did you have a history of thyroid disease or thyroid disfunction?

(1) Yes, I was diagnosed as: (2) No

8. Did you have any history of chronic disease?

(1) Yes, I was diagnosed as: (2) No

9. Have you ever smoked since you are pregnant? (1) Yes (2) No

10. Your home address is city county/district town/street

11. Are you taking any vitamins or iodine-containing supplements at present?

(1) Yes (2) No (Go to Part II)

12. What is the brand name?

①αLNA-RunKang ②21 Super-Vitamins

③Blackmores ④ Elevit (Made in Australia)

⑤ Materna ⑥ Mead Johnson milk powder

⑦ One a day ⑧ Orthomol Natal

⑨ (Please specify: )

##### Part II. Practices

**1. Use of iodized salt**

Did you use salt to cook the main meal eaten by members of your family last week?

(1) Yes (2) No (3) Don’t know/no answer

If Yes:

Please ask the respondent 30g salt sample.

##### Part III. Iodine-related knowledge

1. Is iodine an essential micronutrient for humans?

(1) Yes (2) No (3) Don’t know

2. Does IDD in pregnancy have adverse effects on fetal brain development?

(1) Yes (2) No (3) Don’t know

3. Does IDD in pregnancy have adverse effects on fetal growth and development?

(1) Yes (2) No (3) Don’t know

4. Can IDD be preventable?

(1) Yes (2) No (3) Don’t know

5. What is the most efficient method to prevent IDD?

(1) Iodized salt (2) Seafood (3) Others

6. What does the logo ( **
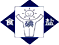
**) on the salt package indicate?

(1) Salt has been iodized (2) Nonsense (3) Don’t know

7. Do you need consuming iodized salt?

(1) Yes (2) No (3) Don’t know

8. Can using iodized salt be replaced with having enough seafood?

(1) Yes (2) No (3) Don’t know

9. Do women need more iodine in pregnancy than non-pregnancy?

(1) Yes (2) No (3) Don’t know

10. Is the current iodine nutrition in Zhejiang pregnant women excessive?

(1) Yes (2) No (3) Don’t know

Code of the salt sample：

Iodine concentration in the salt sample: ppm

Code of the urine sample：

Iodine concentration in the urine sample: μg/L

Name of the investigator (Signature): Investigated Date:
