# Supplementary material for: Poor Iodine Knowledge, Coastal Region, and Non-Iodized Salt Consumption Linked to Low Urinary Iodine Excretion in Zhejiang Pregnant Women
Source: Nutrients. 2019 Feb 15;11(2):413. doi: 10.3390/nu11020413 (PMC6412776; doi:10.3390/nu11020413)
Supplement: Supplementary file 1 [file nutrients-11-00413-s001.zip › Supplementary Materials/Table S1.docx]

**Supplementary materials**

**Table S1. Influential factors of knowledge on low UIC among pregnant women via a binary logistic regression**

| **Parameter** | **β** | ***p*** | **OR (95% CI)** |
| --- | --- | --- | --- |
| Is iodine element essential to humans. (Yes=1; No/Don’t know=0) | **0.338** | **0.005** | 1.402 (1.106‒1.779) |
| Do IDD in pregnancy have adverse effects on fetal brain development? (Yes=1; No/Don’t know=0) | **-0.323** | **0.039** | 0.724 (0.533‒0.984) |
| Do IDD in pregnancy have adverse effects on fetal growth? (Yes=1; No/Don’t know=0) | -0.262 | 0.105 | 0.769 (0.561‒1.056) |
| What is the most efficient method to prevent IDD? (Consuming seafood=1; Consuming iodized salt/Don’t know=0) | **0.702** | **<0.001** | 2.018 (1.527‒2.668) |
| Do you need consuming iodized salt? (Yes=1; No/Don’t know=0) | **-0.406** | **<0.001** | 0.667 (0.532‒0.834) |
| Can using iodized salt be replaced with having enough seafood? (Yes=1; No/Don’t know=0) | 0.163 | 0.168 | 1.177 (0.933‒1.484) |
| Is the current iodine nutrition in Zhejiang pregnant women excessive? (Yes=1; No/ Don’t know=0) | 0.129 | 0.236 | 1.138 (0.919‒1.410) |
