# Supplementary material for: Poor Iodine Knowledge, Coastal Region, and Non-Iodized Salt Consumption Linked to Low Urinary Iodine Excretion in Zhejiang Pregnant Women
Source: Nutrients. 2019 Feb 15;11(2):413. doi: 10.3390/nu11020413 (PMC6412776; doi:10.3390/nu11020413)
Supplement: Supplementary file 1 [file nutrients-11-00413-s001.zip › Supplementary Materials/Table S2.docx]

**Supplementary materials**

**Table S2. Influential factors of sociodemographic characteristics, knowledge, and type of salt consumed on low UIC among pregnant women via a generalized linear model**

|  |  |  | **95% Wald Confidence Interval** | |  |
| --- | --- | --- | --- | --- | --- |
| **Parameter** | **β** | **Std. Error** | **Lower** | **Upper** | ***p*** |
| Residency (rural=1, urban=0) | 0.003 | 0.027 | -0.05 | 0.056 | 0.925 |
| Employment (employed=1, unemployed=0) | -0.013 | 0.0287 | -0.069 | 0.044 | 0.661 |
| Education (high school and above=1, others=0) | -0.025 | 0.029 | -0.082 | 0.032 | 0.387 |
| Income (high income=1, others=0) | -0.013 | 0.0406 | -0.093 | 0.066 | 0.745 |
| Type of salt consumed (Iodized=1, non-iodized=0) | -0.128 | 0.0577 | -0.241 | -0.015 | **0.027** |
| Total knowledge scores (≥28=1, others=0) | 0.241 | 0.0533 | 0.137 | 0.346 | **0** |
| Region (coast=1, land=0) | 0.131 | 0.0305 | 0.071 | 0.191 | **0** |
